# An Innovative Way to Model Twitter Topic-Driven Interactions Using Multiplex Networks

**DOI:** 10.3389/fdata.2019.00009

**Published:** 2019-06-06

**Authors:** Obaida Hanteer, Luca Rossi

**Affiliations:** Data Science and Society Lab, IT University of Copenhagen, Copenhagen, Denmark

**Keywords:** multiplex networks, multiplex community detection, thematic communities, thematic clusters, thematic multiplex, social network analysis, social media data analysis

## Abstract

We propose a way to model topic-based implicit interactions among Twitter users. Our model relies on grouping Twitter hashtags, in a given context, into themes/topics and then using the multiplex network model to construct a thematic multiplex where each layer corresponds to a topic/theme, and users within a layer are connected if and only if they used the same hashtag. We show, by testing our model on a real-world Twitter dataset, that applying multiplex community detection on the thematic multiplex can reveal new types of communities that were not observed before using the traditional ways of modeling Twitter interactions.

## 1. Introduction

The unprecedented amount of data that is produced, on a daily base, on social media has provided to researchers and practitioners a new opportunity to study, in depth, complex social dynamics at a large scale. Within this context, Twitter can easily claim the award for the most researched social media platform. Thanks to the large user-base and a relatively generous API policy, this micro-blogging platform has quickly evolved into the *de-facto* standard platform for multiple studies on social media dynamics.

The detection of cohesive subgroups in social networks, also called as community detection, has been perceived as one of the most valuable tools to better understand social networks (Papadopoulos et al., 2012). Given that members of the same community tend to share some properties, the community structure of a network can provide a better understanding of the overall functioning of this network. The application of this on social media data has provided useful insights about some of the dynamics and phenomena that take place in such systems (Silva et al., 2017).

A common approach to model Twitter interactions for community detection tasks is to build a network based on following/follower relations (Kwak et al., 2010), or networks based on either retweets (Conover et al., 2011) or explicit mentions indicated by the @ character (Yang and Counts, 2010). Advances on multiplex community detection have suggested that looking at more than one of these types of connections together can provide some insights that cannot be observed by looking at each of them separately. As to the content generated by Twitter users, it has been mostly used for topic detection tasks (Ibrahim et al., 2018) and sentiment analysis (Ceron et al., 2014). To the best of our knowledge, no previous work has addressed extracting network-like information from the content generated by users on social media platforms for community detection tasks.

Much of Twitter contemporary interactions happen in the form of conversations in many-to-many polyadic spaces defined by hashtags (Bruns and Burgess, 2011). In this type of interactions, Twitter users are not necessarily retweeting, replying to, or mentioning each other but engaging directly with specific issues. This suggests that analyzing Twitter data by considering only the direct interactions among users (i.e., following/follower, retweet, and mention networks) is still far from providing a complete picture of Twitter-based interactions. In this paper, we address this gap by proposing an innovative way to model topic-driven interactions of Twitter users using the multiplex network model (Dickison et al., 2016). We test our model, the thematic multiplex, on a real-world dataset capturing the Twitter interactions of the Danish politicians during the parliamentary elections of 2015. We show that detecting communities on the thematic multiplex can reveal different dynamics than those observed by analyzing only explicit interactions. For example, we observed, using thematic multiplex community detection, that while some themes/topics were discussed by almost all the parties within the month leading to the election day, left and right-wing parties, at the same time, have also focused on themes that were politically closer to their traditional ideologies.

The rest of this paper is organized as follows. In section 2 we introduce the thematic multiplex and the thematic multiplex community detection. This is followed by our analysis of a real-world use case (section 3) which captures the Twitter interactions among Danish politicians during the parliamentary elections of 2015. We discuss our results in section 4 and conclude our findings in section 5

## 2. The Thematic Multiplex

**Figure 1:**
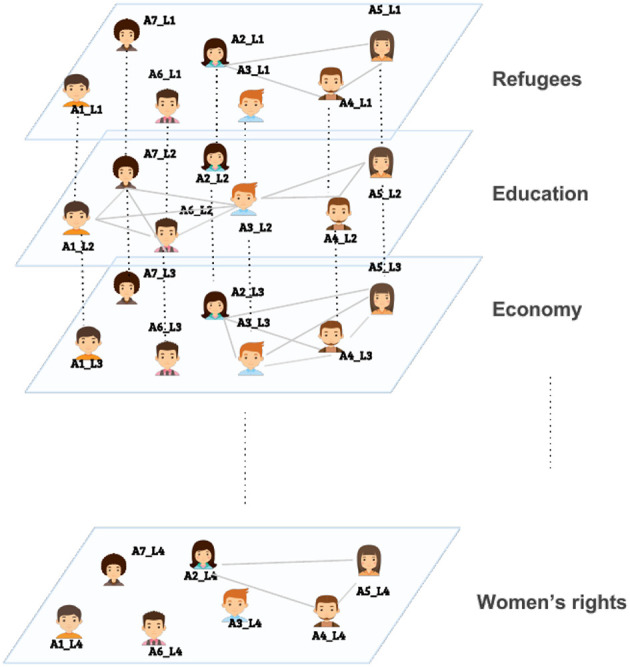
An example of a thematic multiplex.

**Figure 2:**
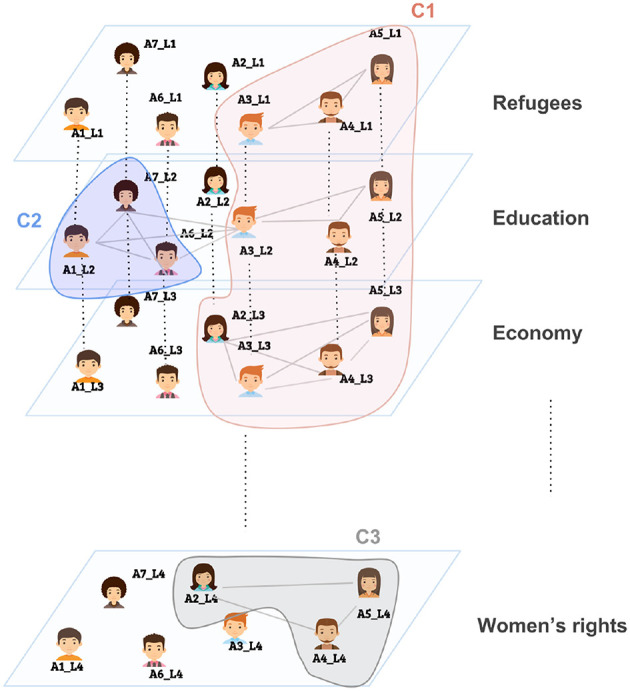
A possible output for applying community detection on a thematic multiplex.

On platforms like Twitter, when a user uses a specific hashtag in a tweet, he/she is not only increasing the visibility of that tweet, but also implicitly, even if not directly, communicating with other Twitter users who are using the same hashtag. This concept has been referred to as the *imagined audience* in the literature (Litt, 2012). Thus, we can assume a social tie (an edge) between two users who used the same hashtag and this is the main idea behind the thematic multiplex. The thematic multiplex, as the name suggests, is a multiplex network where each layer corresponds to a topic/theme and users within a layer are connected via a clique, if and only if, they used the same hashtag. An edge among two actors in the resulted thematic multiplex does not necessarily imply a direct interaction among them yet it suggests that they share a topical-interest. Figure 1 illustrates a thematic multiplex where each layer represents a specific topic/theme (for example, refugees, education, etc.), and users who used the same hashtag within a topic are connected via a clique, which might result in multiple cliques within a layer (for example, the education theme). Figure 2 illustrates a possible output for community detection on the thematic multiplex.

We claim that detecting communities on the thematic multiplex network using multiplex community detection can reveal different dynamics than those observed by analyzing the direct interactions among users. The reason is two folded: on one side, direct interactions are often driven by heterogeneous behavior from the users, e.g., Retweets can represent a form of endorsement or just a way to spread an information deemed to be relevant, Replies can equally be produced by amused conversations or endless fights between users. On the other side, direct interactions are just part of the whole Twitter data, thus any approach focusing solely on those will loose potentially relevant information. Thematic multiplex community detection, on the opposite, results in thematic communities were users are grouped together if they tend to discuss/be involved in the same topics/themes through direct or indirect interactions. More over, given that the qualitative analysis is added in the modeling phase, this intrinsically contributes to the qualitative power of community detection on the thematic multiplex network.

## 3. A Case Study

We describe the dataset in section 3.1, then we discuss the construction of the correspondent thematic multiplex and some choices for our analysis tools in section 3.2. We report our observations on the results in section 3.3.

### 3.1. The DkPol Dataset

The data we use to test our model is collected during the month leading to the 2015 Danish parliamentary election. Starting from a list of all the Danish politicians running for the parliament who also had a Twitter account, we collected all the tweets written during the 30 days leading to the election. The initial dataset was formed by 490 politicians distributed across 10 parties, 5,985 original tweets, 633 replies, and 3,993 retweets. Together with their Twitter activity, we noted also the political affiliation of the 490 politicians. Given the complexity of the Danish multi-party system, the parties have also been grouped into two main coalitions existing at the time: Red Block, currently at the opposition, and the Blue block, currently in government^1^. In order to use the hashtag contained in the tweets to build a thematic multiplex, some initial data cleaning was necessary. The hashtags were first qualitatively analyzed. We then excluded the hashtags that were just about the election campaign as such (like #dkpol) and those referring to political TV debates (like #tv2valg and #DRdinstemme). After this filtering we were left with only 23 hashtags used to refer to specific topics (12 topics). Table 1 shows the grouping of these hashtags into topics. While our suggested grouping can be further discussed as hashtags can be grouped in many other ways, we chose to keep our focus on the correspondent thematic multiplex and the resulted communities for the sake of this paper.

**Table 1:** The main themes discussed on Twitter by the danish politicians during the parliamentary elections of 2015.

	**Theme**	**Hashtag**
1	Children	#dajegvar12
2	Climate	#dkgreen – #talklima – #verdensvildesteforskel
3	Economy	#talop – #dkain – #socialdumping – #nulv
4	Education	#skolechat – #uddpol
5	Election's Practices	#nypolitiskkultur
6	Europe	#eurdk
7	Government Interference	#frihed
8	Health	#sundpol – #sundhed
9	IT	#itpol – #itvalg
10	Refugees	#nuloverdeigen – #engangvarjegflygtning
11	Woman's Rights	#100aaret
12	Work	#arbejde – #dksocial – #dagpenge

### 3.2. Experimental Settings

**Figure 3:**
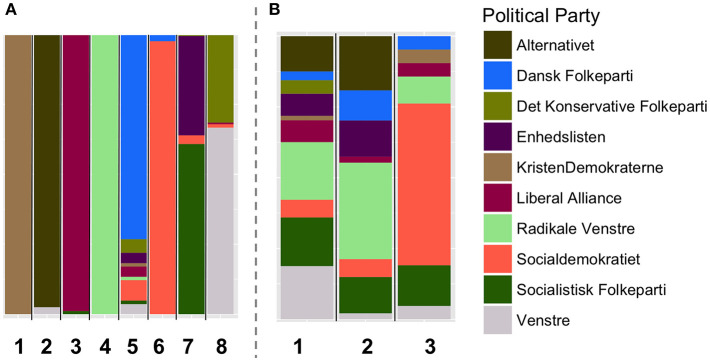
The resulted communities by applying community detection using Generalized Louvain on two different multiplex networks over the DkPol dataset: **(A)** the multiplex constituted of the three layers (following/follower, retweet, and reply) and **(B)** the thematic multiplex. Each bar refers to a different community and the colors in each bar (i.e., community) refer to the composition of each community in terms of the political affiliation of the members constituting it.

**Figure 4:**
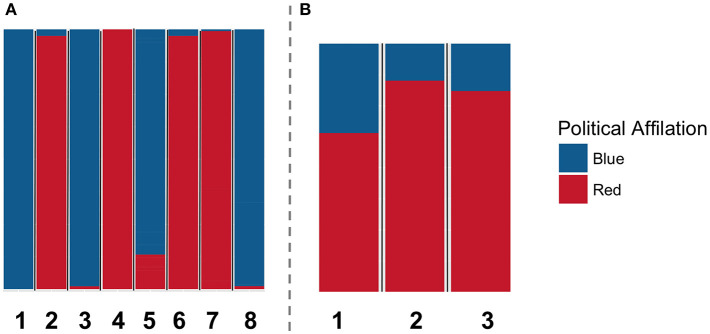
The resulted communities by applying community detection using Generalized Louvain on two different multiplex networks over the DkPol dataset: **(A)** the multiplex constituted of the three layers (following/follower, retweet, and reply) and **(B)** the thematic multiplex. Each bar refers to a different community and the colors in each bar (i.e., community) refer to the composition of each community in terms of the political coalition (red, blue) of the members constituting it.

Given the DkPol dataset, we constructed a twelve-layer thematic multiplex (layer per theme/topic). A topic/theme with *k* hashtags is interpreted as *k* cliques in the correspondent layer (a clique per hashtag) among all the users who used the same hashtag. We first show that detecting communities on the thematic multiplex reveals communities that are largely different from those detected using the traditional ways of modeling twitter interactions. Figures 3, 4 illustrate the communities detected on the multiplex constituted of the following/follower layer, the retweet layer and the reply layer (A), and those detected on the thematic multiplex (B). The two solutions are largely different in terms of the number of detected communities (8 in the first multiplex, and 3 in the second one), and the composition of each community in terms of the political coalition and the political affiliation of the members constituting each community.

As to the selection of the community detection method for our multiplex networks in this paper, we chose a modularity-maximization based community detection method, Generalized Louvain (Jutla et al., 2017) for this task. The reason is that we consider, by assumption, our networks to be undirected networks and our initial focus is on analyzing the communities resulted by the structural features of the network rather than the information flow. For that reason, we chose Generalized Louvain given that it is a well referenced method in the literature to detect this type of communities. The method define communities by optimizing the modularity of the network. In simple graphs, i.e., one layer networks, this translate to finding the best partitioning of nodes into groups, i.e., communities, that maximize the amount of edges within these groups and minimize the number of edges among them. As to the multi-layer extension of this method, it finds the best partitioning that maximize the multi-layer modularity function which is an extension of the simple modularity defined for simple networks. The extended version of modularity introduces a new parameter to the modularity function that is the coupling parameter ω among nodes that belong to the same actor (i.e., the same Twitter user in our case). When ω = 1 (the default case), this means that the coupling among nodes that belong to the same actor is strong. As a result, a partitioning where multiple nodes that belong to the same user (a node represents the existence of a user in a specific layer) lie in the same community contributes intrinsically to the final score of the extended-modularity. In the rest of this paper, we will refer to the output of a community detection method (which is a set of communities) as a clustering.

To better understand the topical dynamics during the month leading to the elections, we chose to create 4 thematic multiplex networks (one for each week content during the month leading the election day). The reason behind choosing “1 week” as a time-window based on which we split the data is that during the month leading to the elections, politicians had to debate on a public TV show once per week.

As illustrated in Figure 2, the resulted communities do not necessarily expand over all the layers, meaning that some topics can be absent in some communities. In addition, nodes may not be evenly distributed over layers (for example, community *C*_1_ in Figure 2 is constituted of 3 nodes in each of the Refugees layer and the Education layer and 4 nodes in the Economy layer). This suggests that topics have different weights, and as a result priorities, in each community which can be interpreted as: some communities, for example, discuss the topic Economy more intensely than they do with the topic Education. To clearly illustrate this, we construct a bipartite network from each clustering. The goal from these bipartite networks is to visualize the relationship between the communities of each clustering and the topics. The width of an edge in the bipartite network between a community and a topic reflects the extent to which that topic is prioritized in that community.

**Figure 5:**
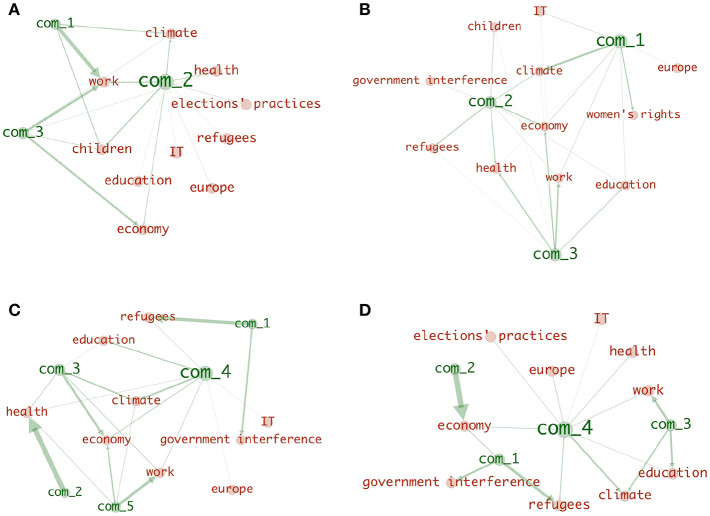
The relationship between the topics and the thematic communities resulted by applying community detection on 4 thematic multiplex newtorks that captured the Twitter interactions of Danish politicians during the month leading the parliamentary lections of 2015 (one per week). **(A)** week 1, **(B)** week 2, **(C)** week 3, **(D)** week 4.

**Figure 6:**
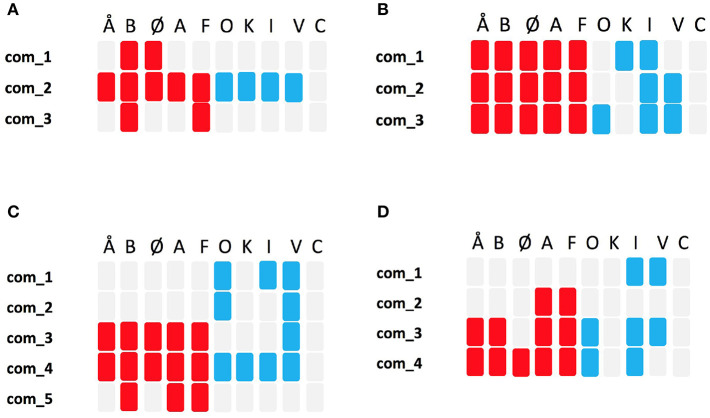
The compositions of the communities reported in Figure 5 in terms of their political affiliation/party and political coalition/block. **(A)** week 1, **(B)** week 2, **(C)** week 3, **(D)** week 4.

Figure 5 shows the resulted bipartite networks, one per week. We invite our reader to look at this figure together with Figure 6 which reports , in the form of colored mini-tables , the composition of each community in terms of political coalitions. The existence of a party in a community is represented as a colored cell in the relevant column in that table. The color of that cell can either be red (if the party is from the red Block) or blue (if the party is from the blue block). A cell that is neither blue nor red implies the absence of that party (identified by the correspondent column) in the community identified by its row.

### 3.3. Observations

By looking at Figure 5A together with Figure 6A, we see that applying community detection on the thematic multiplex of the first week resulted in three communities. Two communities, com_1,com_3, that focus more on economic issues (economy theme and work theme) are composed solely of left-wing (red block) parties. In addition, one community, com_1, constituted of almost all the red block and the blue block parties, tackled all topics with more focus on children, climate, work, and economy themes. Only one of the 12 themes (woman's rights) is absent in all the online debates happened with the first week. The analysis of Figures 5A, 6A shows how during the first week of the election campaign there was a set of bipartisan topics, that were deemed to be central and worth debating, from both political blocks and other themes that were part of political messages of only one of the two blocks.

This scenario seems to change during the second week as Figure 5B together with Figure 6B report the absence of single-coalition communities. However, the differences among the communities can be observed on the level of their topical interests. For example, com_1 has more focus on woman's rights and climate issues, com_2 equally prioritized refugees, health and economy issues, while com_3 had focused on work, education, work, and economy. It is also interesting to observe how some of the topics that were, during the previous week, part of a single coalition community (e.g., “economic issues” in com_3 during the first week but part of a bipartisan community - com_2 - in the second week), are now part of the bipartisan conversation. While the detailed study of this dynamic process is outside the goal of this paper, this seems to suggest that opposite coalition might follow each others' themes in order to be present in the topical debate.

During the third week can observe a new polarization of the picture. Figures 5C, 6C show, com_1, com_2, constituted of only blue block parities with interests in refugees, government interference, and health issues. One community, com_5, is constituted of only red block parties with interests on economical issue (work and economy themes). One community, com_3, constituted of almost mostly red block parties (with only one blue block party) with interests in both climate and economy. A debate among almost all parties is still present in the third week represented by com_3 with more focus on climate. These topical division seems very much aligned with the core political values of the two blocks at the time of the election.

This topical difference is largely maintained into the fourth week, the week of election, where we can see—Figures 5D, 6D—four thematic communities. Com_1 which is constituted of only right-wing parties (blue block) with interests in refugees and government interference issues, com_2 which is constituted of only left wing parties (red block) with interests only in economy, and both com_3, com_4 which are mixed in terms of the coalitions, and with main interests in (work/education/climate) and climate, respectively.

## 4. Discussion

A clear difference has been shown when analyzing the communities on the thematic multiplex versus those detected on a multiplex constituted of the following/follower, retweet, and reply layers. This strongly suggests that community detection on the thematic multiplex reveals different dynamics than those observed using traditional ways of modeling twitter interactions. This is not to say that the thematic multiplex can substitute the traditional ways of modeling Twitter activities, but just to shed a light on different dynamics that can be observed using this way of modeling.

Applying longitudinal community detection on the thematic multiplex network obtained from Twitter data allowed us to observe several interesting dynamics. Given that the dataset captured the interactions among Danish politicians during the month leading the parliamentary elections of 2015, we were able to capture the interest of a political party (or coalition) in specific issues, regardless of the fact that the issue produced an explicit interaction with other users through retweets or replies. During a political campaign, when much of the communication is aimed at promoting the party's agenda to the potential voters, which does not necessarily involve retweeting or replying actions , this type of implicit communication is of key importance. Nevertheless, the thematic multiplex network approach was also able to observe the topics that were more contentious between the parties as well as the topics highly polarized. Moreover, the combination between multiplex thematic analysis and longitudinal data allowed us to show how the political debate, and resulting political communities, are highly dynamic and driven by the ongoing events or campaign themes.

While there might exist other ways to model topic driven implicit interactions on Twitter for clustering tasks, we still think that using multiplex network model offers clear advantages. First, the multiplex network model is a well-developed and widely used model for modeling complex systems (Cardillo et al., 2013; De Domenico et al., 2015) and therefore, provides a powerful, and at the same time flexible, modeling tool that allows for translating properties and variables of complex systems into multi-layer graph proprieties. Second, the plethora of community detection methods developed to detect communities in multiplex networks provides practitioners with more power to choose what works the best for the context of their data.

The idea of moving the qualitative analysis to the modeling phase in the thematic multiplex adds lots of power to the interpretability of the output of a community detection task on this multiplex network. While a fully automated approach to group hashtag into themes/topics could seem a tempting idea, the real complexity behind social media hashtagging is still far from being fully understandable by natural language processing tools and text mining technologies currently at hand. An example are two of the hashtags in our collection: *#engangvarjegflygtning* (translated: *one day I was a migrant*) and *#dajegvar12* (translate: *when I was twelve*). In both cases an *emotional* hashtag is used to discuss specific issues, the refugee crisis with the first and children policies with the latter. The connection between the topic and the hashtag is not explicit, and while both hashtags are clearly topical hashtags (thus referring to a specific topic or event and suggesting the desire of the user to participate to an ongoing larger conversation Bruns and Moe, 2014) they also contain an emotional layer that, as well as the specific topic, is hard to understand if taken out of the specific cultural and societal context.

A future iteration on this work should consider testing the thematic multiplex on other datasets. An important extension should also consider the scalability problem with large scale datasets. The main complexity of this model comes from the greedy approach of connecting the user with his imagined audience via a clique. This means that by using a hashtag for only one time, a user is adding to the model a number of edges equals to the number of all other users who used the same hashtag. While a naive approach to minimize the size of these cliques could be to apply a threshold on the number times a user should use a hashtag before being part of the clique, we still think that further research should be carried out to find other alternatives for the clique concept in the thematic multiplex without any loose in the information.

Even though the idea of using hashtags to gather communications of users that are not otherwise connected (e.g., not following each other) was originally introduced by Twitter, many other platforms such as Facebook and Instagram have adopted this idea in various ways. Thus, we suggest that this model should not be limited to Twitter data as it could be easily applied to other hashtag-based communicative contexts (e.g., Instagram) as well as to other conceptually similar digital contexts (e.g., participation in Facebook pages).

On a separate note, we would like to mention the fact the resulted communities may largely depend on the chosen community detection method. Indeed, weather or not the thematic communities will be significantly different among different community detection methods can be a research question on its own and we think that answering this question is out of the scope of this paper.

## 5. Conclusion

In this paper we propose an innovative model, the thematic multiplex, to model topic-driven interactions on Twitter. The thematic multiplex is a multi-layer network where each layer corresponds to a different topic, and users (nodes) within a layer will be connected via a clique if and only if they used the same hashtag. We explain the motivation behind the thematic multiplex which is the fact that it considers implicit interactions among users on Twitter that are usually neglected in other models. We construct the thematic multiplex of a real-world Twitter dataset describing the Twitter interactions among the danish politicians during the parliamentary elections of 2015. We show that applying multiplex community detection on the thematic multiplex allows us to observe different dynamics than those we would observe on other models.

## Data Availability

The datasets analyzed for this study can be found in the dkpol GitHub repository on the following link [https://github.com/obaidaITU/dkpol].

## Author Contributions

OH and LR conceived of the presented idea and developed the theory, discussed the results, and contributed to the final manuscript. OH performed the experiments. LR supervised the findings of this work.

### Conflict of Interest Statement

The authors declare that the research was conducted in the absence of any commercial or financial relationships that could be construed as a potential conflict of interest. The handling editor and reviewer (SG) declared their involvement as co-editors in the Research Topic, and confirm the absence of any other collaboration

## Footnotes

^1^The red block coalition groups the following parties: Alternativet, Radikale Venstre, Enhedslisten, Socialdemokratiet, and Socialistisk Folkeparti, while the blue block coalition groups: Dansk Folkeparti, KristenDemokraterne, Liberal Alliance, Venstre, and Det Konservative Folkeparti.

## References

[B1] Bruns A. Burgess J. E. (2011). The use of twitter hashtags in the formation of *ad hoc* publics, in Proceedings of 6th European Consortium for Political Research General Conference (Reykjavik: University of Iceland), 1–9.

[B2] Bruns A. Moe H. (2014). Structural layers of communication on twitter, in Twitter and Society, Vol. 89, eds Weller K. Bruns A. Burgess J. Mahrt M. Puschmann C. (New York, NY: Peter Lang), 15–28.

[B3] Cardillo A. Gómez-Gardeñes J. Zanin M. Romance M. Papo D. del Pozo F.. (2013). Emergence of network features from multiplexity. Sci. Rep. 3:1344. 10.1038/srep01344 23446838 PMC3583169

[B4] Ceron A. Curini L. Iacus S. M. Porro G. (2014). Every tweet counts? How sentiment analysis of social media can improve our knowledge of citizens' political preferences with an application to Italy and France. New Media Soc. 16, 340–358. 10.1177/1461444813480466

[B5] Conover M. Ratkiewicz J. Francisco M. R. Gonçalves B. Menczer F. Flammini A. (2011). Political polarization on twitter, in Proceedings of the Fifth International Conference on Web and Social Media (AAAI Press), 89–96.

[B6] De Domenico M. Nicosia V. Arenas A. Latora V. (2015). Structural reducibility of multilayer networks. Nat. Commun. 6:6864. 10.1038/ncomms7864 25904309

[B7] Dickison M. E. Magnani M. Rossi L. (2016). Multilayer Social Networks. Cambridge: Cambridge University Press.

[B8] Ibrahim R. Elbagoury A. Kamel M. S. Karray F. (2018). Tools and approaches for topic detection from twitter streams: survey. Knowl. Inf. Syst. 54, 511–539. 10.1007/s10115-017-1081-x

[B9] Jutla I. S. Jeub L. G. S. Mucha P. J. (2017). A Generalized Louvain Method for Community Detection Implemented in Matlab. Technical report.

[B10] Kwak H. Lee C. Park H. Moon S. (2010). What is twitter, a social network or a news media?, in Proceedings of the 19th International Conference on World Wide Web (Raleigh, NC: ACM), 591–600.

[B11] Litt E. (2012). Knock, knock. Who's there? The imagined audience. J. Broadcast. Electron. Media 56, 330–345. 10.1080/08838151.2012.705195

[B12] Papadopoulos S. Kompatsiaris Y. Vakali A. Spyridonos P. (2012). Community detection in social media. Data Min. Knowl. Discov. 24, 515–554. 10.1007/s10618-011-0224-z

[B13] Silva W. Santana A. Lobato F. Pinheiro M. (2017). A methodology for community detection in twitter, in Proceedings of the International Conference on Web Intelligence (New York, NY: ACM), 1006–1009.

[B14] Yang J. Counts S. (2010). Predicting the speed, scale, and range of information diffusion in twitter, in Proceedings of the Fourth International Conference on Weblogs and Social Media (Washington, DC), 355–358.

